# Dynamic edge-based biomarker non-invasively predicts hepatocellular carcinoma with hepatitis B virus infection for individual patients based on blood testing

**DOI:** 10.1093/jmcb/mjz025

**Published:** 2019-04-08

**Authors:** Yiyu Lu, Zhaoyuan Fang, Meiyi Li, Qian Chen, Tao Zeng, Lina Lu, Qilong Chen, Hui Zhang, Qianmei Zhou, Yan Sun, Xuefeng Xue, Yiyang Hu, Luonan Chen, Shibing Su

**Affiliations:** 1 Institute of Interdisciplinary Integrative Medicine Research, Shanghai University of Traditional Chinese Medicine, Shanghai, China; 2 Key Laboratory of Systems Biology, Center for Excellence in Molecular Cell Science, Institute of Biochemistry and Cell Biology, Shanghai Institute of Biological Sciences, Chinese Academy of Sciences, Shanghai, China; 3 Center for Excellence in Animal Evolution and Genetics, Chinese Academy of Sciences, Kunming, China; 4 Qidong Liver Cancer Institute, Qidong People’s Hospital, Qidong, China; 5 Institute of Liver Disease, Shuguang Hospital, Shanghai University of Traditional Chinese Medicine, Shanghai, China; 6 Minhang Branch, Zhongshan Hospital/Institute of Fudan-Minhang Academic Health System, Minhang Hospital, Fudan University, Shanghai, China; 7 School of Life Science and Technology, Shanghai Tech University, Shanghai, China; 8 Research Center for Brain Science and Brain-Inspired Intelligence, Shanghai, China

**Keywords:** hepatitis B virus, hepatocellular carcinoma, diagnosis and prognosis, edge-based biomarker, dynamic network biomarker

## Abstract

Hepatitis B virus (HBV)-induced hepatocellular carcinoma (HCC) is a major cause of cancer-related deaths in Asia and Africa. Developing effective and non-invasive biomarkers of HCC for individual patients remains an urgent task for early diagnosis and convenient monitoring. Analyzing the transcriptomic profiles of peripheral blood mononuclear cells from both healthy donors and patients with chronic HBV infection in different states (i.e. HBV carrier, chronic hepatitis B, cirrhosis, and HCC), we identified a set of 19 candidate genes according to our algorithm of dynamic network biomarkers. These genes can both characterize different stages during HCC progression and identify cirrhosis as the critical transition stage before carcinogenesis. The interaction effects (i.e. co-expressions) of candidate genes were used to build an accurate prediction model: the so-called edge-based biomarker. Considering the convenience and robustness of biomarkers in clinical applications, we performed functional analysis, validated candidate genes in other independent samples of our collected cohort, and finally selected *COL5A1*, *HLA-DQB1*, *MMP2*, and *CDK4* to build edge panel as prediction models. We demonstrated that the edge panel had great performance in both diagnosis and prognosis in terms of precision and specificity for HCC, especially for patients with alpha-fetoprotein-negative HCC. Our study not only provides a novel edge-based biomarker for non-invasive and effective diagnosis of HBV-associated HCC to each individual patient but also introduces a new way to integrate the interaction terms of individual molecules for clinical diagnosis and prognosis from the network and dynamics perspectives.

## Introduction

Hepatocellular carcinoma (HCC) is predicted to be the sixth most commonly diagnosed cancer and the fourth leading cause of cancer death worldwide in 2018 (Bray et al., 2018); most HCC cases result from liver cirrhosis caused by chronic hepatitis B (CHB) or C virus infection (Yang et al., 2018). Notably, nearly half of new HCC cases come from China, which is also a major area of hepatitis B virus (HBV) infection. An early diagnosis of HCC remains urgent for effective treatment of patients to prevent the progression of HBV-induced HCC and to reduce risk of mortality, especially in the early stage of carcinogenesis.

Currently, the conventional methods for HCC diagnosis include ultrasound, serum alpha-fetoprotein (AFP), computed tomography scanning, and magnetic resonance imaging. Ultrasound is mainly recommended for screening and surveillance; however, it exhibits only a moderate sensitivity of 60% (Singal et al., 2009) and an extreme reliance on operator experience. Although an AFP level of >400–500 ng/ml is considered a gold standard diagnostic criterion for HCC at present, 30% of the patients with a low AFP level are already in an advanced stage (Waidely et al., 2015). These methods have their own limitations in terms of accuracy, sensitivity, and timeliness.

To address these issues, numerous studies have developed and provided novel strategies from different viewpoints, especially for molecular biomarkers (He et al., 2012; Sa et al., 2016; Llovet et al., 2018). Within the bloodstream, peripheral blood mononuclear cells (PBMCs) represent a reservoir of inflammatory cells that contribute to disease progression by different means. It has been repeatedly demonstrated in recent years that genetic expression in PBMCs is altered in the context of malignancy (Baine et al., 2011; Jiang et al., 2016). Some studies have identified characteristic changes (e.g. expression or mutation) of disease-associated genes in PBMCs that could be used for HCC diagnosis, due to the fact that both chronic HBV infection and HCC progression can lead to dysfunctions of the immune system, and PBMCs, as the most common immune cell population throughout the whole body, may play an important role in reflecting these dysfunctions (Shi et al., 2014; Ding et al., 2015; Peng et al., 2016). Recent publications suggest that genes in tumor-educated circulating PBMCs are valuable surrogate markers with diagnostic potential and prognostic applications in different cancer localizations such as lung (Zhou et al., 2016), colorectal (Ganapathi et al., 2014), breast (Suzuki et al., 2018), and digestive cancers (Honda et al., 2010; Kenneth Wayne et al., 2010). These findings have provided remarkable evidence for developing blood-based tests for HCC diagnosis. However, most of these studies focused on individual and static biomarkers (i.e. node-based or vertex-based biomarkers), which mainly contribute to HCC diagnosis or prognosis based on static and low-dimensional characteristics (e.g. by using single markers with corresponding thresholds according to their different concentrations). Considering the complicated and personalized pathogenesis of HBV-induced HCC, it is necessary to introduce a new way to integrate the interaction effects of individual molecules into clinical diagnosis and prognosis from the perspectives of network and dynamics—the so-called edge-based biomarker (Zeng et al., 2016). Different from the traditional node-based biomarker, the edge-based biomarker requires significant differences of correlations between two biomolecules (e.g. genes and proteins) rather than their individual concentrations to predict the specific stage during disease progression for individual patients (Zhang et al., 2014; Liu et al., 2016a; Yu et al., 2017). The high-dimensional information included in an edge-based biomarker can achieve accurate diagnosis and prognosis, even with individual heterogeneity. In addition, another advantage of an edge-based biomarker is that it can provide clues on pathological mechanisms during disease progression or functional activity (i.e. gain or loss) responses to therapeutic regimens (Zeng et al., 2016).

**Figure 1:**
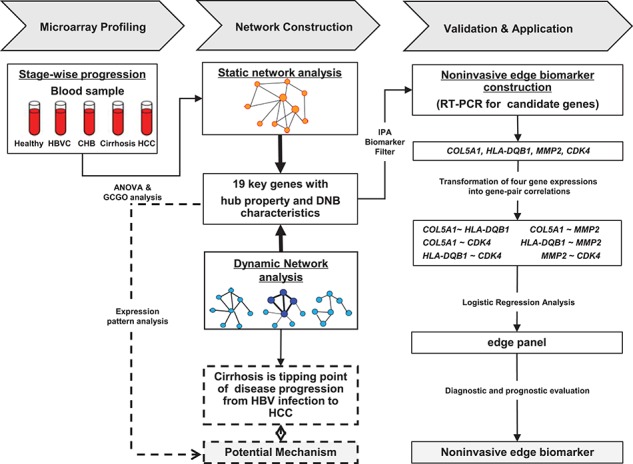
Study flowchart of identifying edge-based biomarkers. Candidate genes were roughly filtered by ANOVA and gene profile clustering with gene ontology analysis (GCGO). Next, we created static correlation networks across all disease stages to identify hub genes for further selecting candidate molecular signatures with network information and then tested these genes with dynamic characteristics based on the three criteria of DNBs, which can predict and signal the critical transition before hepatocellular carcinogenesis. To build the predictive edge-based model with the selected genes, we transformed their gene expression levels (vertex space in math) into gene-pair correlations (edge space in math) based on each single sample data point (Zeng et al., 2016). Finally, a multiple logistic regression model with forward selection techniques was used to build a baseline risk model. Three edges (gene pairs) were retained to form the edge panel, which was significantly associated with an HCC diagnosis in the model.

In this study, to identify novel edge-based biomarkers for specific and non-invasive diagnosis of HCC with HBV infection for individual patients, we collected blood samples from both healthy donors and patients with chronic HBV infection in different states (i.e. HBV carrier (HBVC), CHB, cirrhosis, and HCC). By analyzing the transcriptomic profiles of PBMCs with our developed algorithm at a network level (Figure 1), we identified 19 candidate genes whose properties satisfied dynamic network biomarkers (DNBs) (Chen et al., 2012). After functional analysis and further selection, *COL5A1*, *HLA-DQB1*, *MMP2*, and *CDK4* were considered as key genes due to their robustness in an independent cohort. Then, we integrated the interaction terms of the individual genes to build prediction models. Actually, our edge-based biomarkers could be considered to specifically distinguish HCC stage and to further effectively predict outcomes for individual patients.

## Results

### Dynamic transcriptomic profiles of PBMCs from patients in different stages were measured during HBV-associated HCC and healthy donors

We recruited 306 clinical participants, including healthy donors and patients in different disease states according to the progression of HBV-associated HCC (i.e. HBVC, CHB, cirrhosis, and HCC), according to the corresponding criteria of inclusion and exclusion (Supplementary Table S1), while their clinical information (Supplementary Table S2) and blood samples were collected in a methodical and systemic way (details shown in Methods). Then, we randomly chose blood samples from 3 healthy donors as well as 3 HBVC, 10 CHB, 10 cirrhosis, and 10 HCC patients from the previous cohort to measure the transcriptomic profiles of their PBMCs.

**Figure 2:**
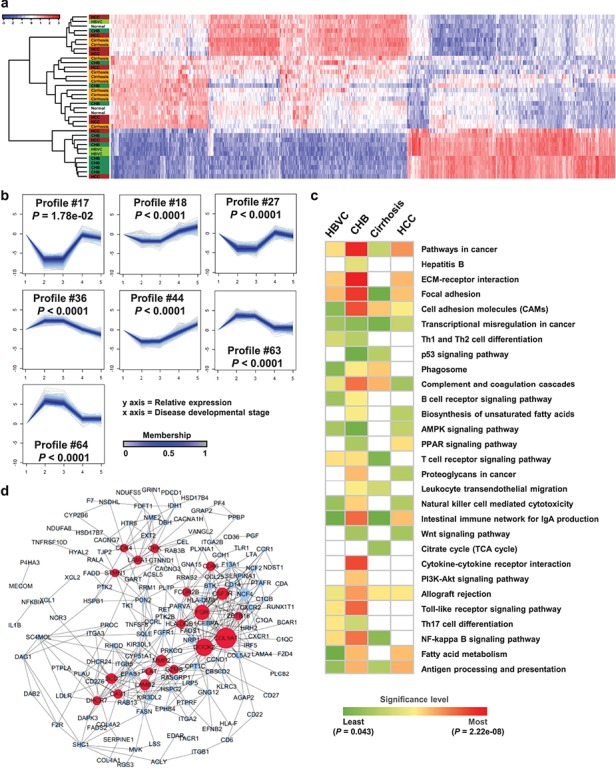
Gene profile cluster analysis and cross-stage network construction from HBV infection to HCC. (**a**) Unsupervised hierarchical clustering was performed to distinguish different stages based on 1906 DEGs. (**b**) Seven unique gene profile clusters (#17, #18, #27, #36, #44, #63, and #64) were identified using cluster analysis of gene expression dynamics according to the time-series gene expression data. The horizontal axis represents different disease stages from healthy to HCC, and the vertical axis represents the gene expression value after log-normalized transformation. The *P*-value indicates significance. (**c**) Functional phenotyping of active genes with the acquisition or loss of co-expression edges in the static network across stages of HCC development. (**d**) The static subnet shows 19 signature genes (red nodes) with the highest degrees, consisting of 159 nodes (genes) and 421 edges (gene pairs). The size of each node represents the degree value.

A total of 1906 differentially expressed genes (DEGs) (Supplementary Table S4) were identified by a random variant model (RVM) *F*-test with a corresponding *P-*value of <0.05 after FDR correction. Then, we performed unsupervised hierarchical clustering (Figure 2a) based on these DEGs to characterize different stages during the progression of HBV-associated HCC from the normal. Unfortunately, samples at different stages could not be clearly and independently distinguished by hierarchical clustering, except for some of samples at cirrhosis. This result might imply that using molecular signatures at the individual expression level might make it difficult to represent complicated changes in a systematic and organized manner during the initiation and evolution of a complex disease.

**Figure 3:**
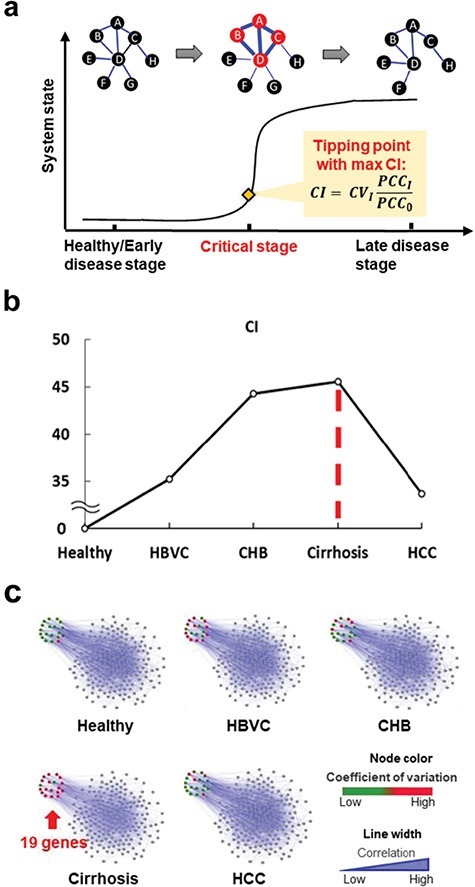
A total of 19 signature genes with dynamic characteristics satisfying the 3 criteria of DNB. (**a**) Brief description of the three criteria of DNB theory. Specifically, near the tipping point, DNB members have a high coefficient of variation (*CV*), the PCC among members of DNB (*PCC_i_*) increases, and the average PCC between the DNB members and others (*PCC_o_*) decreases. (**b**) DNB analysis on 19 signature genes from healthy to HCC disease states. The critical stage was identified as cirrhosis, with a max *CI* score. (**c**) Networks graphically illuminate the dynamic changes of the 19 signature genes in the network structure.

**Figure 4:**
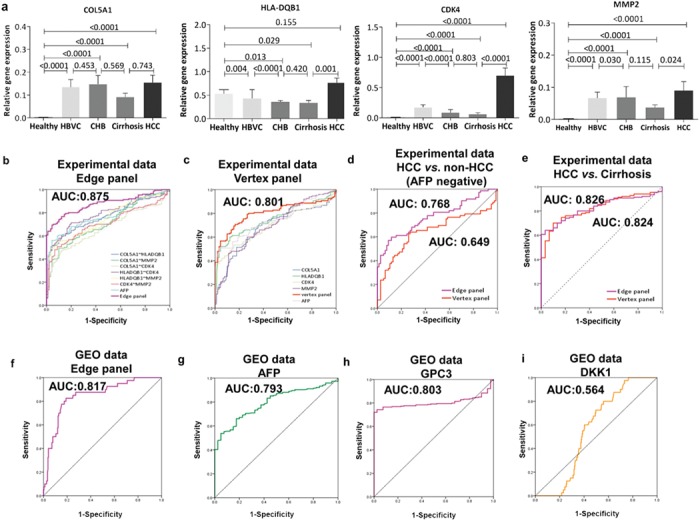
Diagnostic capacity assessments for the four core candidate genes. (**a**) RT-PCR validation of *COL5A1*, *HLA-DQB1*, *CDK4*, and *MMP2* in blood samples of other independent participants from our cohort. The vertical axis represents GAPDH-normalized relative expression values and their expression differences among stages during the progression of HBV-associated HCC measured by Mann–Whitney *U* tests. (**b–e**) ROC curve analysis of the logistic panel, individual genes, and panels in AFP-negative (AFP of <20 ng/ml) patients (*n* = 72 out of 150 HCC samples; *n* = 77 non-HCC samples), HCC vs non-HCC patients and cirrhosis patients vs HCC patients. (**f–i**) Comparison of the edge panel, the traditional biomarker AFP, and the new proposed biomarkers GPC3 and DKK1 in the GEO data set GSE25097 by ROC curve analysis.

**Figure 5:**
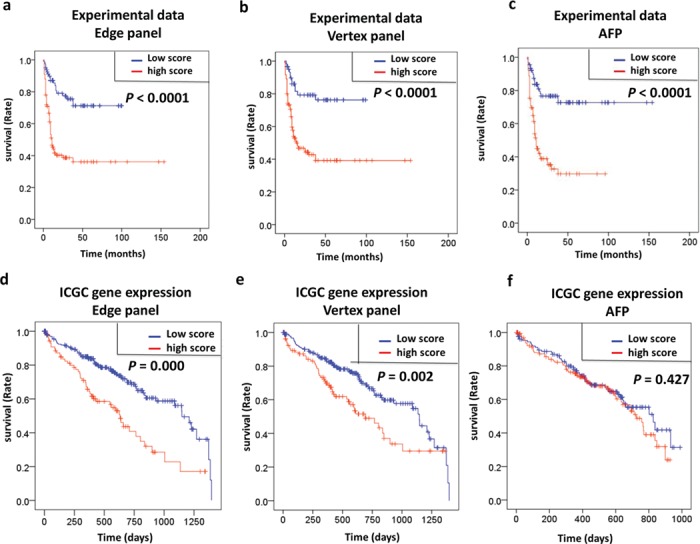
Kaplan–Meier plot analysis and log-rank test survival analysis. (a–c) Negative associations between the vertex panel, edge panel, and AFP with overall survival were noted for HCC patients (all *P* < 0.001, log-rank test) in an experimental data set. (d–f) Negative associations were found between the vertex panel and edge panel with overall survival (all *P* < 0.001, log-rank test) on our experimental data and independent ICGC data, but the AFP mRNA level did not show a prognostic capacity for HCC patients in the ICGC data set.

### Stage-characterized candidate biomarkers were identified by static network analysis

To address the above challenge on the poor performance of distinguishing stages by molecular signatures at the individual expression level, we introduced biomarkers with network information. Generally, a complex disease results from dysfunctions, not in individual molecules but via a systematic interplay of multiple molecules and even biological functions (Barabási and Oltvai, 2004; Elkington, 2006). To obtain non-invasive biomarkers to precisely and specifically distinguish HCC stages from other disease stages during the progression of HBV-associated carcinogenesis, we developed an algorithm to identify stage-characterized biomarkers based on network information (Figure 1).

First, based on the changing trends in expression between two consecutive stages, we clustered the DEGs into corresponding profile patterns by cluster analysis of gene expression dynamics (Ramoni et al., 2002; Rmann, 2003) (Supplementary Table S5). A total of 7 out of 80 total clusters were selected with statistical significance (*P* < 0.05) (Figure 2b; members of each cluster are listed in Supplementary Table S6).

Then, we constructed a static network weighted by pair-wise correlations among members of the seven significant profile clusters to estimate their functional activity at a comprehensive interactome level during the progression of HBV-associated HCC (details shown in Methods). To investigate the mechanisms of HCC carcinogenesis, we performed functional analyses on active genes with the acquisition or loss of co-expression edges in a stimulus-dependent manner [56] across stages of HCC development. As shown in Figure 2c, typical HCC-associated pathways (e.g. PI3K-Akt, p53, and Wnt signaling pathways) were identified (Tian et al., 2016; Debebe et al., 2017), and their dynamic activities implied the complicated regulations during disease progression. In particular, KEGG pathways associated with cell metabolism and communications (e.g. focal adhesion, cell adhesion molecules, complement and coagulation cascades, steroid biosynthesis, and transcriptional misregulation in cancer) were significantly and abnormally active across all stages of HBV-associated carcinogenesis; six related to the immune response (i.e. Th1 and Th2 cell differentiation, B cell receptor signaling pathway, cytokine–cytokine receptor interaction, toll-like receptor signaling pathway, Th17 cell differentiation, and chemokine signaling pathway) were enriched only in stages of HBV infection and CHB (Lara-Pezzi et al., 2001). Similarly, results of GO analysis showed that dysfunctions in HBV infection and CHB are mainly involved in fatty acid/lipid oxidation and regulation of cell proliferation, while dysfunctions in HCC initiation and development are mainly involved in regulation of the Wnt signaling pathway (Supplementary Table S7 and Figure S1). These findings might suggest that complicated dysfunctions in immune responses from HBV infection to final HCC were involved in a failure of anti-tumor immunity and an altered tumor microenvironment (Marshall et al., 2013).

Generally, hub molecules have been considered to play important regulatory roles in conducting biological functions and maintaining stability of a network. Thus, we extracted 19 hub genes with the highest degrees as candidate biomarkers (Figure 2d). These signature molecules presented stage-characterized dynamics during the progression of HBV-associated HCC (Supplementary Table S8); furthermore, their involved biological functions and processes were significantly related to human hepatic dysfunctions and hepatocarcinogenesis (Supplementary Figure S2). These functional analysis results showed that these candidate biomarkers played different and complicated roles during the progression of HBV-associated HCC.

### The candidate molecular signatures satisfied the three criteria of DNBs

Traditionally, molecular biomarkers for disease diagnosis or prognosis are mainly based on their static information (e.g. different concentrations), which cannot provide early-warning signals at the tipping point or critical period (i.e. pre-disease state) during disease progression. To address this issue for early diagnosis, DNBs were introduced based on the three criteria from the non-linear dynamic theory (e.g. fluctuation changes of concentrations of leading molecules, dynamic associations, or correlations among leading molecules) to predict malignant phase transition during disease initiation and deterioration (Chen et al., 2012). Here, we tested whether these 19 candidate molecules have dynamic characteristics according to the three criteria of DNBs. The DNB method has been applied successfully to studies in multiple fields (e.g. disease diagnosis and prognosis, therapeutic response, and cell differentiation) (Mojtahedi et al., 2016; Richard et al., 2016; Lesterhuis et al., 2017; Li et al., 2017b; Yang et al., 2018). Specifically, if a system approaches the critical phase transition, a leading group of indicative molecules (i.e. DNBs) appear and satisfy the following three conditions: (i) drastic increases in fluctuation for molecular concentrations, which can be evaluated by the coefficient of variation (*CV*); (ii) increases in correlations among molecules in this leading group, which can be evaluated by Pearson correlation coefficients (PCCs) (*PCC*_i_); and (iii) decreases in correlations between molecules in this leading group and others, which can be evaluated by PCCs (*PCC*_o_). A criterion index (*CI*) is provided as a numerical signal by comprehensively integrating the three parameters (*CV*, *PCC*_i_, and *PCC*_o_) of the DNB method. When *CI* reaches the peak across the measured stages, the corresponding stage is considered the critical period of the biological system (Figure 3a).

After calculating the *CIs* according to expression data of these candidate molecules in each stage during HCC progression, we found a strong signal of critical stage before hepatocarcinogenesis at cirrhosis (Figure 3b), which is considered as the tipping point. This result is consistent with the liver morphological alterations during the initiation and evolution of HBV-associated HCC (Wang et al., 2002; Sung et al., 2008). As shown in Figure 3c, the series of networks graphically demonstrated the dynamic changes in the network structure and expression variations of these candidate biomarkers. This result also confirmed that the 19 signature genes possessed both network features (hubs) and dynamic features (DNBs) of HCC progression that not only characterized disease progression but also provided early-warning signals of disease deterioration.

### The candidate signatures were validated in other independent samples of the clinical cohort

In clinical practice, biomarkers are expected to provide accurate predictions and to be convenient on operation. Therefore, it is necessary to further select core typical markers from the previous 19 candidate molecules. After literature mining and Ingenuity Pathway Analysis (IPA) biomarker searching, 8 out of 19 molecules were further selected and confirmed to have significant relationships to hepatic diseases (Supplementary Table S9). Then, to obtain the robust signatures for predicting the progression of HBV-associated HCC, we validated the expression profiles of these 8 candidate molecules across all stages of disease progression in other independent samples of the clinical cohort, which includes 30 healthy donors as well as 30 healthy, 30 HBVC, 30 CHB, 30 cirrhosis, and 150 HCC samples. Finally, four out of the eight genes (*COL5A1*, *CDK4*, *MMP2*, and *HLA-DQB1*) were used for the further diagnosis and prognosis of HBV-associated HCC due to their stage-characterized signatures and remarkable consistency in expression levels in other independent samples (Figure 4a; Supplementary Figure S3).

### The edge-based biomarkers achieved better performance in diagnosis

Traditionally, molecular biomarkers (i.e. node-based biomarkers) contribute to HCC diagnosis mainly according to individual and static information (e.g. differential expression). However, considering the complicated and personalized pathogenesis of HBV-induced HCC, edge-based biomarkers with the interaction effects of individual molecules (e.g. pair-wise correlations of molecules) were expected to improve biomarker performance in terms of precision, sensitivity, and specificity. Here, we created predictive models via logistic regression based on individual gene expression values (vertex panel) and co-expressions of gene pairs (edge panel) (details shown in Methods).

According to the expression levels of the last four candidate biomarkers measured by quantitative RT-PCR in the independent samples, we found that the diagnostic performance of the edge panel was better than individual gene pairs, even for the typical AFP (Figure 4b; Supplementary Table S10) as with the vertex panel (Figure 4c; Supplementary Table S10). Obviously, the edge panel had its own advantages in HCC diagnosis in AFP-negative patients (Figure 4d; Supplementary Table S10). Meanwhile, these identified molecules with dynamic information were expected to predict the critical transition to HCC for early diagnosis. Thus, we respectively used the vertex panel and the edge panel to distinguish cirrhosis from other stages during the progression of HBV-associated HCC and found that the edge panel showed better performances in indicating cirrhosis, which was considered as the critical stage of malignant transition to HCC (Figure 4e; Supplementary Table S10). To test the generality and superiority of our edge panel for HCC diagnosis, we collected another independent data set from the Gene Expression Omnibus (GEO) (GSE25097), which included 268 HCC patients, 40 cirrhosis patients, and 6 healthy samples, and we compared diagnostic performance of our edge panel (Figure 4f) with the traditional biomarker AFP (Figure 4g) and the new biomarkers GPC3 (Filmus and Capurro, 2013) (Figure 4h) and DKK1 (Shen et al., 2012) (Figure 4i) by receiver operating characteristic (ROC) curve analysis and found that our edge panel had higher discriminatory power (Figure 4f). These findings suggested that our edge panel might be useful in clinical practice based on blood testing, not only to non-invasively distinguish HBV-associated HCC and non-HCC but also to signal imminent carcinogenesis for early diagnosis.

### The edge-based biomarkers can also be used for prognosis

Diagnostic biomarkers are also expected to be used for prognosis (e.g. AFP) (Trevisani et al., 2001; Qin and Tang, 2002). Here, we performed Kaplan–Meier and log-rank test survival analyses to assess the correlations of the vertex and edge panels with overall survival in HCC patients. According to the data from our clinical cohort, the results of the ROC analysis showed that values of the vertex and edge panels were significantly negatively associated with overall survival similar to AFP (all *P* < 0.001; Figure 5a–c), while the vertex and the edge panels had better performances in HCC prognosis than individual genes (Supplementary Figure S4) and gene pairs (Supplementary Figure S5). In addition, we validated our results by using other cohorts (including 298 HCC patients) from the International Cancer Genome Consortium (ICGC) and unexpectedly found that the vertex and edge panels could robustly contribute to HCC prognosis (Figure 5d and e); however, it seemed that AFP lost its power in HCC prognosis (Figure 5f). These results suggested that the vertex and edge panels consisting of the candidate molecules identified by our algorithm could also be a powerful and robust prognostic indicator for HBV-associated HCC.

## Discussion

In this study, we broadly recruited 273 patients suffering from liver diseases (i.e. HBVC, CHB, cirrhosis, and HCC) and 33 healthy donors (Supplementary Table S1). Then, we profiled whole-genome expression levels of PBMCs from blood samples of the above cohort to identify molecular biomarkers for non-invasively diagnosing HBV-associated HCC. After GCGO analysis, 19 candidate genes were found to predict the critical transition stage (i.e. cirrhosis) before hepatocarcinogenesis based on the 3 criteria of DNBs (Figure 3). Then we narrowed the selection (Supplementary Figure S2 and Table S8) and introduced the edge-based biomarker, considering the complicated and personalized pathogenesis of HBV-induced HCC (Figure 1). We selected 4 core genes (*COL5A1*, *HLA-DQB1*, *MMP2*, and *CDK4*) from the 19 candidates to form the node/edge panel after performing literature mining and expression validation in independent cohorts (Figure 4a; Supplementary Figure S2). Interestingly, following our algorithm, most candidate signatures belonged to well-known cancer hallmarks (Hanahan and Weinberg, 2011).

Although the complex process of hepatocarcinogenesis is still not fully understood, several signal pathways have been identified as critical players in the pathophysiology of HCC, including the Wnt/β-catenin pathway, the p53 pathway, and so others (Aravalli et al., 2008). The alterations in these pathways may differ at the same time, which is probably the cause of the insufficient sensitivity of single biomarkers. The possibility to perform gene profiling in combination with systems biology approaches has led to a new era in biomarker development using multiple genes (Schütte et al., 2015). In our study, though *COL5A1*, *HLA-DQB1*, *CDK4*, and *MMP2* are related to different functions in complex pathways of hepatocarcinogenesis, they formed a promising biomarker panel with diagnostic and prognostic capacities.

For each gene in this panel, studies have indicated relationships with carcinogenesis. The *COL5A1* gene is a member of the clade B fibrillar collagen gene family and is involved in the regionalization of fibril-associated macromolecules that are necessary for tissue-specific regulation of the later fibril growth and matrix assembly stages (Wenstrup et al., 2004). Meanwhile, the type V collagen assembled by *COL5A1* may mediate interleukin products and further regulate immune responses, which play especially important roles in auto-immunity (Yamada et al., 2009; Sullivan et al., 2017). Another possible mechanism is *COL5A1*’s involvement in extracellular matrix (ECM) formation (An et al., 2015). *CLO5A1* has been verified to be correlated with several types of cancer (Fan et al., 2016; Zhao et al., 2016; Li et al., 2017a; Liu et al., 2018). *COL5A1* may play an oncogene role, as it can promote cell proliferation, invasion, and survival (Liu et al., 2018). In our study, *COL5A1* expression in cirrhosis was higher than in CHB, which indicated that *COL5A1* might play important roles in the initiation and activation of liver fibrosis as well as the regulation of immune responses (Figure 4). In most organs, the principle components of the ECM are collagens and numerous other proteins that make up the basement membrane. The MMP family has been implicated in the ECM degradation associated with tumor growth and angiogenesis, one of the earliest stages of tumor progression (Lamoreaux et al., 1998; Fang et al., 2000). *MMP2* has been studied as a potential diagnostic or prognostic biomarker of colorectal and ovarian cancers (Hilska et al., 2007; Périgny et al., 2008). Up-regulated *MMP2* expression is often detected in solid tumor tissues and is associated with tumor metastasis in HCC (Tang et al., 2016). Interestingly, matrix metallopeptidase 2 encoded by *MMP2* can mediate type V collagen degradation (Veidal et al., 2012; Van Doren, 2015) and can also initiate an innate immune response with activation of the pro-inflammatory pathways by regulating inflammatory cytokines and chemokines (Nissinen and Kahari, 2014). Overactive cyclin protein in cancer cells often leads to uncontrolled proliferation. Cyclin-dependent kinase 4, encoded by *CDK4*, might play key roles in the tumorigenesis (Wang et al., 2017) of a variety of cancers through deregulation of the CDK4/6–cyclin D–INK4–RB pathway (Graf et al., 2010). In addition, *CDK4* might control cell cycle progression by inducing and maintaining cytokine responsiveness in lymphocytes (Modiano et al., 2000). In our study, *CDK4* expression was significantly increased in the HCC stage (Figure 4). This result is consistent with previous studies, which also reported overexpression of *CDK4* in many tumor types (Lindberg et al., 2007; Poomsawat et al., 2010), suggesting that *CDK4* is a key factor in promoting the initiation and development of tumors. HLA class II molecules have a relevant role in the inflammatory response elicited by most infections. Human *HLA-DQB1* belongs to the HLA class II beta chain paralogs that are expressed in APCs and plays a central role in the immune system by presenting peptides derived from extracellular proteins, which has been recommended for the diagnosis and prognosis of HBV-associated liver diseases (Doganay et al., 2014; Liu et al., 2016b).

To summarize the discussion, the potential mechanisms by which *COL5A1*, *HLA-DQB1*, *CDK4*, and *MMP2* can predict disease stage could include the following: (i) these genes are closely connected to key mechanisms of hepatocarcinogenesis, including immunity (*HLA-DQB1*), ECM-induced tumor formation (*COL5A1* and *MMP2*), and cancer cell proliferation (*CDK4*); (ii) unlike the conventional method of biomarker hunting, these genes were selected from continuous disease stage data with both static and dynamic characteristics instead of comparing HCC vs healthy group only; and (iii) from the networks and dynamics perspectives, we integrated four genes as edge biomarkers that included high-dimensional information and could achieve accurate early diagnosis for HCC.

After determining the core biomarker genes that were significantly associated with the pathogenesis and progression of HBV-induced HCC, we introduced the edge-based method to build an edge panel by logistic regression with integrating interaction terms (e.g. co-expression) among signature molecules instead of a traditional model of diagnosis or prediction by using the vertex panel with a composite of expression levels or concentrations of individual molecules. As a remarkable advantage, edge-based biomarkers can predict phenotypes in an accurate manner, even though their individual genes may have no significantly different expression profiles. As expected, we found better performances of the edge panel in both diagnosis (Figure 4) and prognosis (Figure 5) for HCC than in the vertex panel and the typical biomarkers (e.g. AFP). In particular, the edge panel had a clear advantage for HCC diagnosis in patients without significant increases in AFP (Figure 4d), which implied that our edge-based biomarker could have wider applications in the clinic.

In summary, we recruited specific participants (including HBVC, CHB, cirrhosis, and HCC patients as well as healthy donors) to identify non-invasive biomarkers of HCC. The identified candidate genes at both the network and dynamic levels could not only characterize the progression of HBV-associated HCC but also identify cirrhosis as the critical transition stage before carcinogenesis according to the mathematical model of DNBs. Meanwhile, we introduced edge-based biomarkers with differential correlation/network information instead of traditional differential gene/protein expression information to build the prediction model, which demonstrated that the edge panel had better performances in both the diagnosis and prognosis in terms of both precision and specificity for HCC, especially for patients with AFP-negative HCC, than individual genes and gene pairs as well as the vertex panel. Thus, our study not only provided a novel edge-based biomarker for non-invasive and effective diagnosis of HBV-associated HCC for individual patients based on blood testing but also introduced a new way to integrate the interaction terms of individual molecules for clinical diagnosis and prognosis from the network and dynamics perspectives.

## Materials and methods

### Study design and participants

The study design is shown in Figure 1. First, we broadly recruited participants who satisfied the eligibility criteria (Supplementary Table S1) from the Shuguang Hospital, Shanghai University of Traditional Chinese Medicine (TCM), and the Qidong Liver Cancer Institute between August 2012 and December 2014. A total of 306 blood samples from these participants (including 33 healthy donors, 33 HBVC, 40 CHB, 40 cirrhosis, and 160 HCC patients) were collected (Supplementary Table S2). This study was approved by the Official Ethics Committee of the Shanghai University of TCM, and written informed consent was obtained from all participants. The healthy volunteers had no history of liver disease, no viral infections, and no other diseases. All patients were screened by the diagnostic standard referred to as the `Chronic hepatitis B prevention and treatment guidelines’ (Chronic hepatitis B prevention and treatment guidelines (2010), 2011) (Supplementary Table S1). Tumor differentiation was graded using the Edmondson–Steiner grading system. Demographic and clinic-pathological characteristics were collected for all included participants.

In addition, we obtained a data set (GSE25097) from the GEO database (http://www.ncbi.nlm.nih.gov/geo/) and one HCC data set from the ICGC (https://icgc.org/) for our independent assessment of diagnostic or prognostic capacity. The HCC data set from ICGC provided by RIKEN (project code: LIRI-JP) includes 298 donors (Hudson et al., 2010).

### RNA isolation

Leukocytes were isolated from the blood samples by Ficoll-optimized density gradient separation and were frozen at −80°C (Gertler et al., 2003). Total RNA was extracted using a `two-step’ protocol as described previously (Guo et al., 2012). Total RNA was extracted from leukocytes from whole blood using TRIzol^®^ Reagent (Invitrogen) and was frozen at −80°C. A quality control check was carried out using the NanoDrop ND-1000 (NanoDrop).

### Microarray detection

We randomly selected blood samples from 3 healthy donors, 3 HBVC, 10 CHB, 10 cirrhosis, and 10 HCC patients from our clinical cohort to measure the transcriptomic profiles of PBMCs. Total RNA was extracted, and the biotinylated cDNAs were hybridized to the NimbleGen *Homo sapiens* 12x135K Array (Roche, CAT No. A6484-00-01). Raw data were extracted as pair files by the NimbleScan software (version 2.5); the data were considered robustly expressed if the signal-to-noise ratio was >2. The NimbleScan software uses a robust multi-array analysis algorithm that offers quantile normalization and background correction. Probe level and gene summary files were produced. All microarray data were deposited in the GEO (http://www.ncbi.nlm.nih.gov/geo/), accession number GSE114783. The gene summary files were imported into the Agilent Gene Spring Software (version 11.0) for further analysis. An expression signal cutoff level was set at 50.0 as the minimum number of falsely called probes. Only genes with a signal value above the cutoff level were used in the subsequent analyses.

### Data processing

We selected DEGs according to a random variance model-corrected analysis of variance (ANOVA). An RVM *F*-test is commonly used to filter DEGs because it can effectively increase the degrees of freedom in cases with small sample sizes (Wright and Simon, 2003). These values were FDR-adjusted *P*-values and were considered significant if they were <0.05. All data analyses were performed using the statistical software R version 3.1.0 (http://www.R-project.org).

### Gene profile clustering combining gene ontology analysis (GCGO)

In accordance with the different signal density change tendencies of genes under different situations, we identified a set of unique model expression tendencies. The raw expression values were converted into log_2_ ratios. We defined unique gene profile clusters by using cluster analysis of gene expression dynamics (Ramoni et al., 2002; Rmann, 2003) according to the time-series gene expression data. The significance of each profile pattern was estimated by Fisher's exact test and a multiple comparison test. Additionally, GO analysis was used for function detection for genes of each cluster with specific expression tendencies to uncover potential HCC-associated biological functions enriched by these identified genes.

### Static network

To further select candidate signature genes with network information for distinguishing different stages during hepatocellular carcinogenesis, we constructed a static network with *z*-transformed *PCCs* of gene pairs to obtain the hub genes with high degrees (Barabási and Oltvai, 2004). We considered absolute *PCCs* > 0.9 as strong co-expressions (i.e. links).

### Criteria of DNBs

DNBs were considered to predict the tipping points or critical transitions during disease progression, whose theoretical derivation was demonstrated in our previous work (Chen et al., 2012). Briefly, when a biological system approaches the tipping point or critical state just before the critical transition, the DNB (i.e. a dominant cluster of molecules) would appear and satisfy three criteria as a predictive signal:

(1). The molecules in the cluster are highly fluctuated in terms of their expression levels at the critical stage (i.e. the average coefficients of variation or standard deviations of DNB members are large).

(2). The molecules in the cluster are highly correlated in terms of their expression levels (i.e. the average expression correlation among the DNB members in absolute values is high).

(3). The molecules in the cluster are weakly correlated in terms of expression levels with other molecules (i.e. the average correlation between the molecules of the cluster and other molecule clusters in absolute values is decreased).

Thus, we have the following criterion index *CI* to quantify DNB molecules as well as the tipping point.

$$ CI={CV}_I\frac{PCC_I}{PCC_0}, $$

where *CV_I_* is the average standard deviation of the DNB members, *PCC_I_* is the average PCC of the cluster of molecules in absolute values, and *PCC*_o_ is the average PCC between the cluster of molecules and other molecules in absolute values.

Based on non-linear dynamic theory, when a biological system is near the critical stage or tipping point, a DNB cluster exists and satisfies the above three features. This system will undergo drastic deterioration after the critical stage and further develop to the late disease state (Figure 3a). Thus, the DNB method is able to provide early-warning signals of the critical transition by quantifying DNBs (Chen et al., 2012).

### Predictive models by logistic regression

We created predictive models via logistic regression based on individual gene expression values (vertex panel) and co-expressions of gene pairs (edge panel) as follows.

(i) Vertex (gene) panel. In the logit model based on the four vertices (genes) combined with AFP,

logit (*P* = HCC) = −0.998 + 1.42 × *COL5A1* + 1.278 × *HLA-DQB1*−0.816 × *CDK4* + 0.051 × *MMP2* + 0.019 × AFP.

(ii) Edge (gene-pair) panel. After stepwise logistic regression selection, three edges (gene pairs), including all four genes, were enrolled in the edge panel. In the logit model based on the three edges combined with AFP, we have

logit (*P* = HCC) = −1.714-6.892 × (*COL5A1*~*CDK4*) + 2.088 × (*HLADQB1*~*CDK4*) +7.267 × (*CDK4~MMP2)* + 0.029 × AFP.

### Real-time RT-PCR

RNA samples were qualified for further processing if the A260/A280 spectrophotometric ratio was between 1.8 and 2.1. A total of 1 mg of total RNA was transcribed into cDNA in a 20 μl reaction volume. The primers used are listed in Supplementary Table S3. Each real-time RT-PCR in a final volume of 25 μl contained 2× SYBR Green Real-Time RT-PCR Master Mix, 0.4 μm primers, and 0.5 μl of template cDNA. The cycling conditions consisted of an initial single cycle of 5 min at 95°C, followed by 40 cycles of 30 sec at 95°C, 30 sec at 54°C, 15 sec at 72°C, and fluorescence acquisition at 83°C for 1 sec. The cDNA was synthesized using reverse transcriptase (TOYOBO), oligo (dT), and random primers with 5 μg of RNA from the same samples used in the microarray. The PCR amplifications were performed in duplicate for each sample. The gene expression levels were quantified relative to the expression of β-actin by employing an optimized comparative Ct (ΔCt) value method. The differences in gene expression levels between groups were compared using Student's *t*-test. A *P*-value of <0.05 was considered significant.

### Statistical analyses

Differences in gene expression levels between groups were compared using the Mann–Whitney *U* test. A *P*-value of <0.05 was considered significant. A stepwise logistic regression model was used to combine diagnostic markers. The predicted probability of being diagnosed with HCC was used as a surrogate marker to construct the ROC curve. The area under the ROC curve (AUC) was used as an accuracy index to evaluate the diagnostic performance (Hanley and McNeil, 1983). We defined the cutoff value according to the best predictive values calculated by the ROC analysis, where the Youden's index (sensitivity + specificity − 1) was maximal. Survival probabilities were calculated using the Kaplan–Meier method, and differences in survival between two patient groups were determined by the log-rank test.

### Functional analysis

Genes were subjected to GO analysis (www.geneontology.org/) to uncover their biological processes and molecular functions. Similarly, KEGG pathway analysis (www.genome.jp/kegg/) was used to identify significant pathways associated with the DEGs. We used Fisher's exact test and the ${\chi}^2$ test to select significant GO terms or pathways with a threshold of significance defined by the *P-*value and FDR. Within the significant category of both the GO/pathway analyses, the enrichment Re was given as follows:

$$ \operatorname{Re}=\left({n}_f/n\right)/\left({N}_f/N\right) $$,

where ${n}_f$ is the number of DEGs within the particular category, $n$ is the total number of genes within the same category, ${N}_f$ is the number of DEGs in the entire microarray, and $N$ is the total number of genes in the microarray.

Conventional biomarker filtration analysis of the selected gene set was performed using IPA software (Ingenuity^®^ Systems).

## Supplementary Material

Supplementary_Figures_mjz025

Supplementary_Tables_mjz025

## Notes

**Trial registration:** Identifier: NCT03189992. Registered on June 4, 2017. Retrospectively registered (www.clinicaltrials.gov).
