# Supplementary material for: Dynamic edge-based biomarker non-invasively predicts hepatocellular carcinoma with hepatitis B virus infection for individual patients based on blood testing
Source: J Mol Cell Biol. 2019 Apr 8;11(8):665–77. doi: 10.1093/jmcb/mjz025 (PMC6788726; doi:10.1093/jmcb/mjz025)
Supplement: Supplementary_Figures_mjz025 [file supplementary_figures_mjz025.pdf]

Supplemental materials

Figure S1. Comparisons of enriched GO terms among different stages during HCC development.

Figure S2. Selection of candidate genes and their involved functions.

Figure S3. Validation of gene expressions for the core candidate genes using RT-PCR and microarray analysis.

Figure S4. Kaplan-Meier plot analysis and log-rank test survival analysis of node panel and each gene.

Figure S5. Kaplan-Meier plot analysis and log-rank test survival analysis of edge panel and each edge.

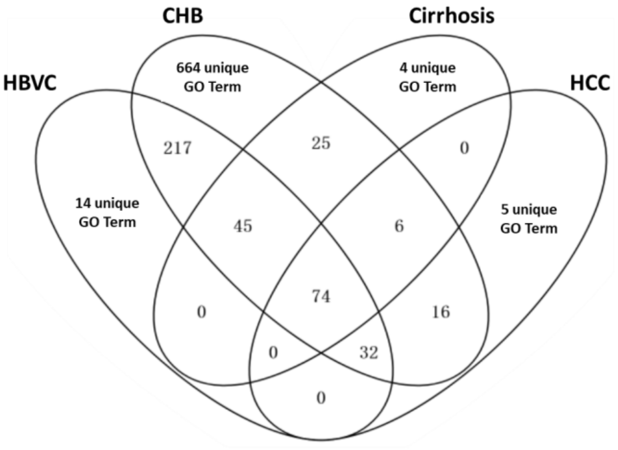

Figure S1. Comparisons of enriched GO terms among different stages during HCC development.

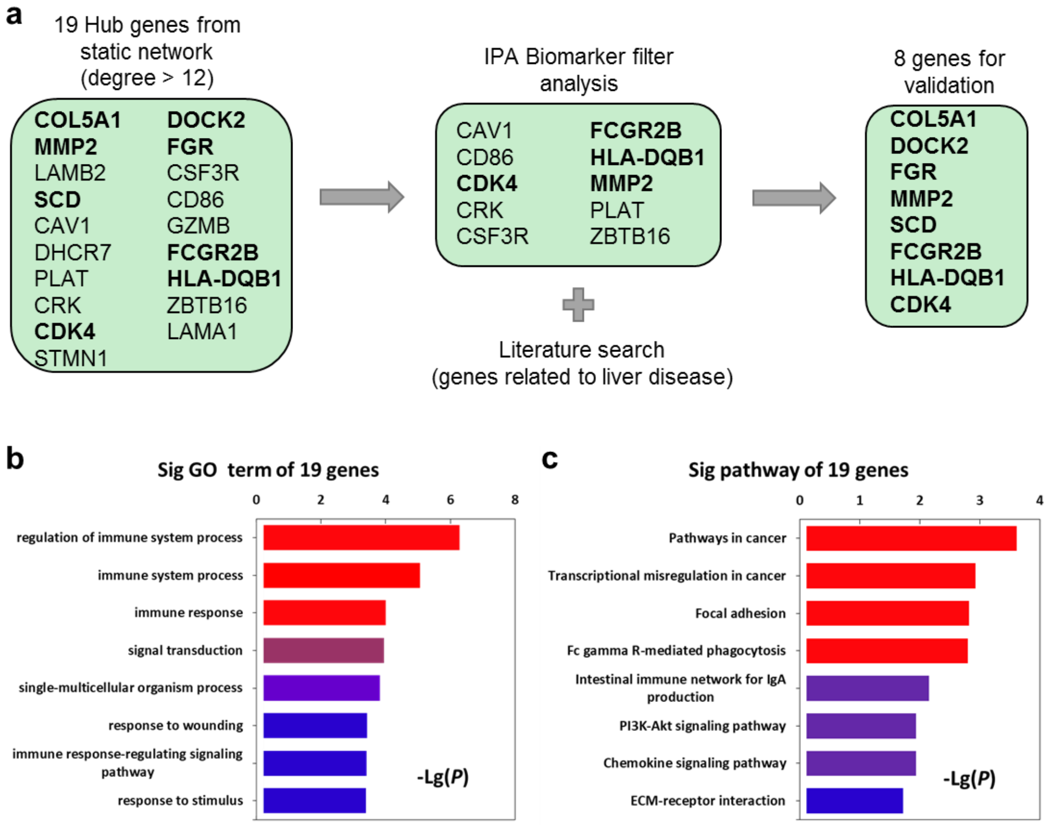

Figure S2. Selection of candidate genes and their involved functions. a Selection of candidate genes. **b** GO analysis and **c** KEGG pathway analysis showed that these candidate biomarkers played different and complicated roles during progression of HBV-associated HCC.

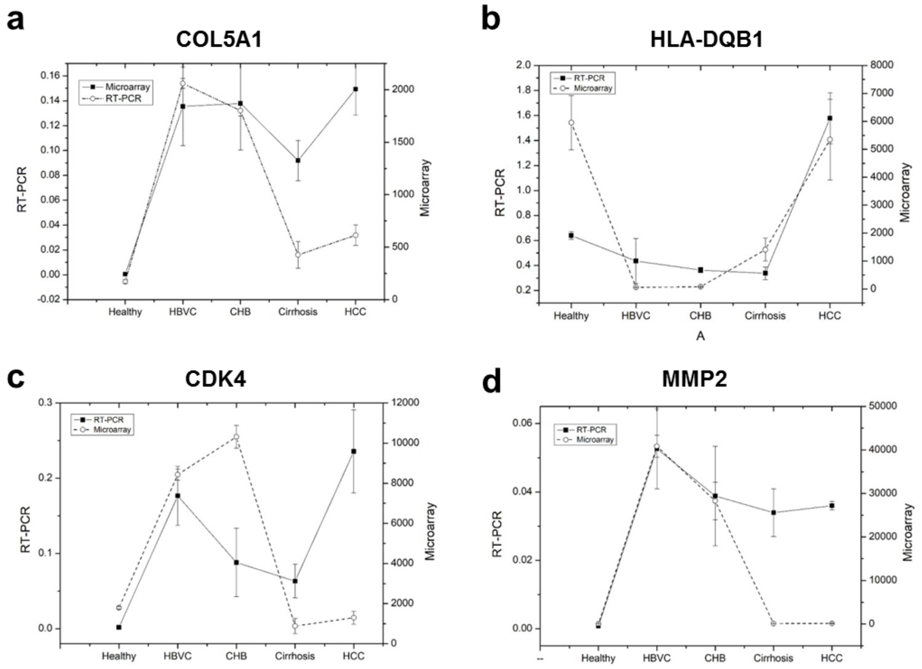

Figure S3. Validation of gene expressions for the core candidate genes using RT-PCR and microarray analysis. a - d Values from the quantitative-PCR are shown on the left *Y* –axis; and values from the microarray are indicated on the left *Y* -axis.

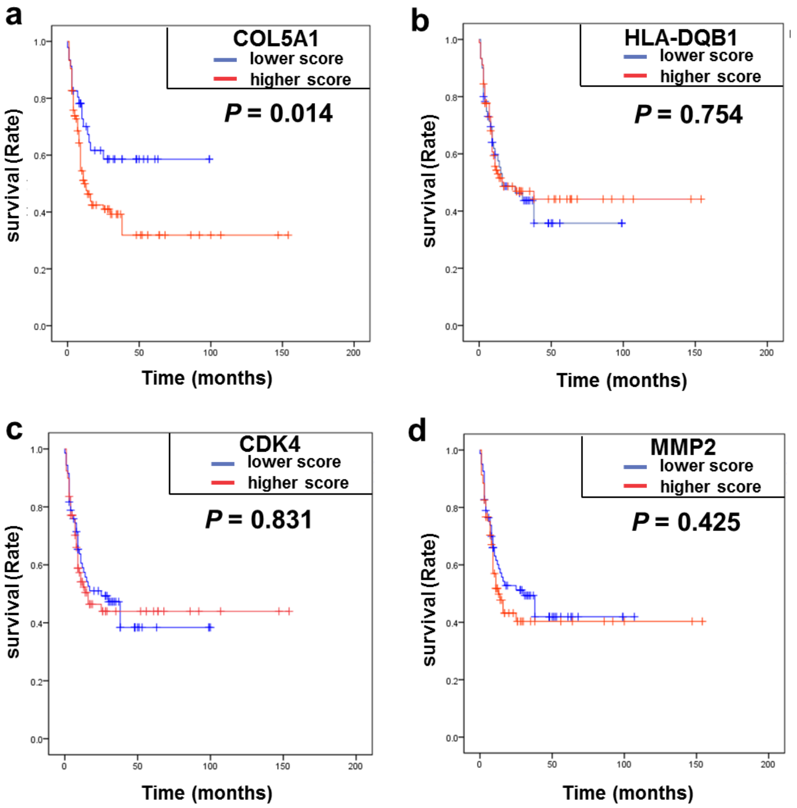

Figure S4. Kaplan-Meier plot analysis and log-rank test survival analysis of node panel and each gene. A negative association between *COL5A1* expression and overall survival was observed for HCC patients (*P*=0.014, log-rank test).

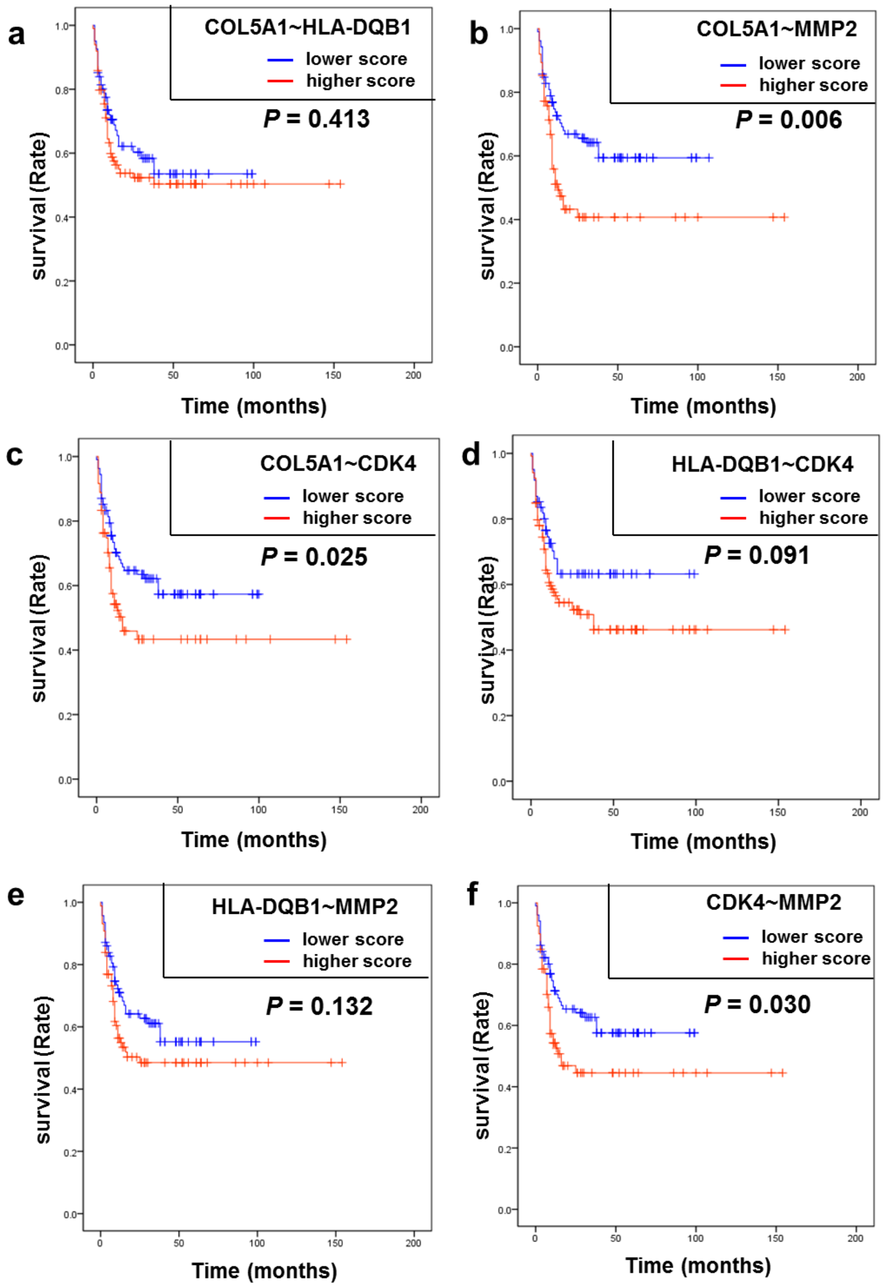

Figure S5. Kaplan-Meier plot analysis and log-rank test survival analysis of edge panel and each edge. A negative association between *COL5A1-MMP2, COL5A1-CDK4,* and *CDK4-MMP2* with overall survival was noted for HCC patients (*P*=0.006, *P*=0.025, and *P*=0.030, respectively, log-rank test).
